# Informational Quality of YouTube Content on Partial Meniscectomy Remains Inadequate

**DOI:** 10.1016/j.asmr.2025.101192

**Published:** 2025-06-02

**Authors:** Shankar S. Thiru, Nicholas E. Aksu, Gregorio Baek, Theodore A. Joaquin, Gregory T. Perraut, William F. Postma

**Affiliations:** a Georgetown University School of Medicine, Washington, D.C., U.S.A.; b Department of Orthopedics, Georgetown University Medical Center, Washington, D.C., U.S.A.

## Abstract

**Purpose:**

To assess the quality of YouTube videos regarding partial meniscectomy.

**Methods:**

The first 50 videos returned by the keyword search “partial meniscectomy” after screening for inclusion and exclusion criteria were included in the study. Off-topic videos, non-English language videos, duplicated videos, YouTube Shorts, and videos with poor audio quality were excluded. The primary outcomes were the DISCERN instrument (range, 15-75), *Journal of American Medical Association* (JAMA) benchmark criteria (range, 0-4), and Global Quality Scale (GQS) (range, 0-5). In addition, date of publication, video duration, number of likes, number of comments, and number of views were recorded. Videos were also categorized by source type (physicians, companies, or patients), subject (surgical technique, patient experience, or overview), and content (educational or subjective patient experience).

**Results:**

Of the 50 videos, 24 (46.0%) were published by physicians; 20 (40.0%), by companies; and 6 (14.0%), by patients. The most prevalent type of information was an overview (44.0%); 86% of the videos were educational in nature, whereas the remaining 14% described subjective patient experiences. The mean video length was 5.07 ± 0.21 minutes. The mean number of views was 1,624,827.44 ± 8,334.86; the mean number of comments, 191.62 ± 34.11; and the mean number of likes, 25,984.84 ± 1,051.76. The mean DISCERN, JAMA, and GQS scores were 45.005 ± 1.75 (95% confidence interval [CI], 44.74-45.49; range, 15-75), 1.83 ± 0.52 (95% CI, 1.68-1.97; range, 0-4), and 2.97 ± 0.52 (95% CI, 2.83-3.11; range, 1-5) respectively. For the JAMA score and GQS score, videos published by physicians had greater quality (both *P* = .01). Finally, overview videos were of the highest quality regarding all scores (*P* < .01 to *P* = .03), whereas educational content had higher quality than patient experience content (*P* < .01).

**Conclusions:**

The overall quality of YouTube videos concerning partial meniscectomy remains poor to suboptimal. Currently, YouTube is not an appropriate resource for orthopaedic patients seeking information about partial meniscectomy.

**Clinical Relevance:**

YouTube is not an appropriate resource for orthopaedic patients seeking information about partial meniscectomy.

Meniscal tears are prevalent orthopaedic injuries that can be addressed with nonsurgical and surgical treatment, such as arthroscopic meniscectomy and meniscal repair.^1^, ^2^, ^3^ Although there has been recent growth and interest in meniscal repair and the associated “save the meniscus” movement, arthroscopic meniscectomy remains a great option for various nonrepairable pathologies.^4^^,^^5^ Thus, partial meniscectomy is still one of the most commonly performed surgical procedures in orthopaedic clinical practice and has favorable postoperative outcomes such as relatively rapid recovery and low morbidity.^6^, ^7^, ^8^

Partial meniscectomy was chosen as the focus of this study because it represents a foundational arthroscopic skill that is frequently performed yet still requires technical proficiency and experience to master. It presents a distinct learning curve, making it especially relevant not only to practicing surgeons but also to trainees and patients seeking to better understand the operation.^8^^,^^9^ Given its prevalence and significance in orthopaedic practice, partial meniscectomy is a commonly searched topic, frequently featured in educational content on platforms such as YouTube (Alphabet, Mountain View, CA).

The past few years have seen a rapid increase in patients seeking medical information on the internet.^10^ Of the existing online platforms, YouTube seems to be one of the primary sources of information on many surgical procedures. Previous studies have shown low-quality information in YouTube videos about anterior cruciate ligament reconstruction, shoulder replacement, rehabilitation after rotator cuff repair, and meniscal root lesions.^11^, ^12^, ^13^, ^14^ Despite this, these videos have been shown to greatly influence patient decision making surrounding surgical management, leading to concern about patient utilization of the resource for medical information.^15^

Although there has been literature assessing the quality of YouTube regarding patient acquisition of health information, to our knowledge, the reliability of YouTube videos regarding partial meniscectomy surgery remains unknown. The purpose of this study was to assess the quality of YouTube videos regarding partial meniscectomy. We hypothesized that the informational quality on YouTube would not meet an adequate standard for patients seeking information on their future meniscal procedures.

## Methods

This was a cross-sectional study that examined videos on partial meniscectomy. This study did not involve human or animal data and required no ethical board approval. To ensure consistency and reproducibility of the video search, all queries were conducted on YouTube using Google Chrome (version 135.0.7049.96; Alphabet) in incognito mode to minimize personalization of results. The following filters and settings were standardized across all searches: the default “relevance” sorting option was used, no additional search filters (e.g., upload date, type, or duration) were applied, and Restricted Mode was turned off. The location was manually set to United States using the settings accessible via the browser’s YouTube interface. The first 50 videos that met the inclusion criteria were selected for analysis based on prior studies indicating that users typically do not engage beyond the initial set of search results, as well as to balance feasibility with comprehensiveness.^16^, ^17^, ^18^ This sample size was chosen to reflect a realistic user experience while maintaining methodologic rigor. The exclusion criteria were off-topic videos, non–English language videos, duplicated videos, YouTube Shorts, and videos with poor audio quality.

**Fig 1:**
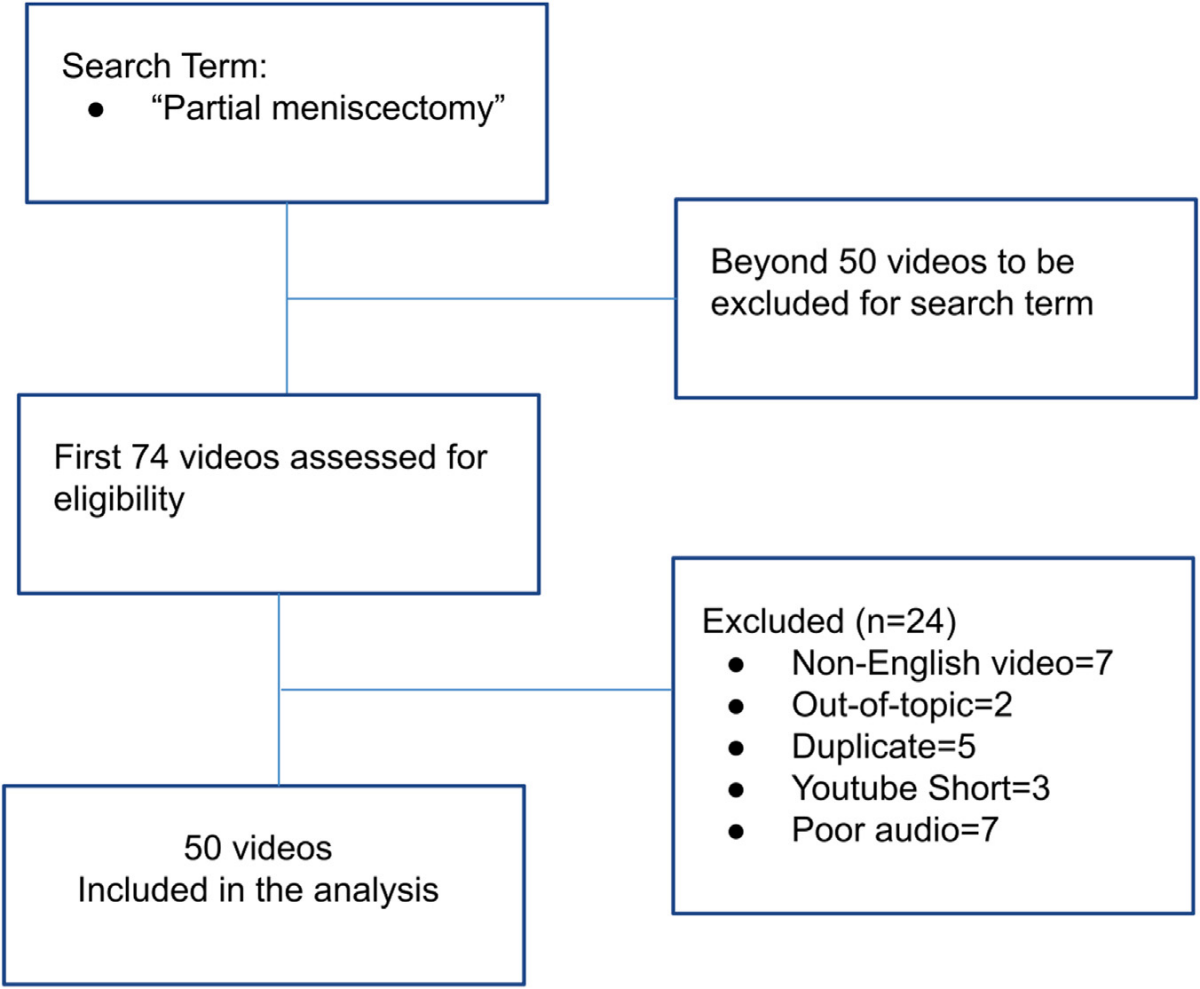
Methodology of selection of YouTube videos for analysis.

Video demographic characteristics such as duration of video, date of publication, number of likes, number of views, and number of comments were collected. Videos were also categorized by source type (physicians, companies, or patients), subject (surgical technique, patient experience, or overview [i.e., videos in which multiple aspects were addressed]), and content (educational or subjective patient experience) (Fig 1). The analysis of video content was independently performed by a junior rater (S.S.T.) and senior rater (N.E.A.) twice, separated by at least 2 weeks, with blinding to prior measurement results. Intrarater reliability (Cohen κ = 0.71) and inter-rater reliability (Cohen κ = 0.65) were found to have substantial agreement and to be suitable for analysis.

### Study Outcomes

The primary outcomes of the study were the DISCERN instrument (range, 15-75), *Journal of American Medical Association* (JAMA) benchmark criteria (range, 0-4), and Global Quality Scale (GQS) score (range, 0-5).

### DISCERN Instrument

The DISCERN instrument is an assessment scale that was created to assess the reliability and quality of informational resources.^19^ The scale involves 16 items in total, which is then divided into 3 parts. The first part consists of items 1 to 8, which measure the reliability of information. Items 9 to 15 make up the second part and measure the quality of information. The final part, item 16, evaluates overall quality rating. Each item uses a 5-point Likert scale to generate a score. For the first 15 items, 1 indicates no and 5 indicates yes, with a range in between. For item 16, 1 indicates “low quality with serious or extensive deficiencies” and 5 indicates “high quality with minimum deficiencies.” Composite DISCERN scores range from 15 to 75. A higher score equates to higher informational reliability and quality. Scoring interpretation is as follows: 15 to 27, very poor; 28 to 38, poor; 39 to 50, medium; 51 to 62, good; and 63 to 75, excellent.^20^

### JAMA Benchmark Criteria

The JAMA benchmark criteria have been one of the primary tools to assess disseminated online health information.^21^ The scale includes 4 criteria: authorship, attribution, currency, and disclosure (Table 1). Scoring interpretation is as follows: 0 to 1, insufficient information; 2 to 3, partially sufficient information; and 4, completely sufficient information.

**Table 1:** Description of JAMA Benchmark Criteria and GQS Scoring

	Description
JAMA criteria	
Authorship	Author and contributor credentials and their affiliations should be provided
Attribution	All copyright information should be clearly listed, and references and sources for content should be stated
Currency	Initial date of posted content and subsequent updates to content should be provided
Disclosure	Conflicts of interest, funding, sponsorship, advertising, support, and video ownership should be fully disclosed
GQS grade	
1	Poor quality and unlikely to be of use for patient education
2	Poor quality and of limited use to patients; some information is present
3	Suboptimal quality and flow; somewhat useful to patients; important topics are missing; some information is present
4	Good quality and flow; useful to patients because most important topics are covered
5	Excellent quality and flow; highly useful to patients

GQS, Global Quality Scale; JAMA, *Journal of American Medical Association*.

### Global Quality Scale

The GQS is a measurement tool that gauges the instructional value of a video for its viewers.^18^ It looks at quality, streaming, and ease of information comprehension. Scoring ranges from 1 to 5, with 1 denoting poorest quality/not useful for viewers and 5 indicating excellent quality/very useful for viewers (Table 1).

### Statistical Analysis

Descriptive statistics were calculated and reported for all video characteristics and scores. Normality of continuous variables was tested, and variables were reported as means with standard deviation or ranges, whereas categorical variables were reported as counts with percentages. Video characteristic associations were tested using the Spearman correlation test. Pair-wise comparisons were performed with a 1-way analysis of variance or its nonparametric counterpart. All statistical analyses were performed with Stata, version 18 (StataCorp, College Station, TX). A 2-tailed *P* value < .05 determined statistical significance.

## Results

**Fig 2:**
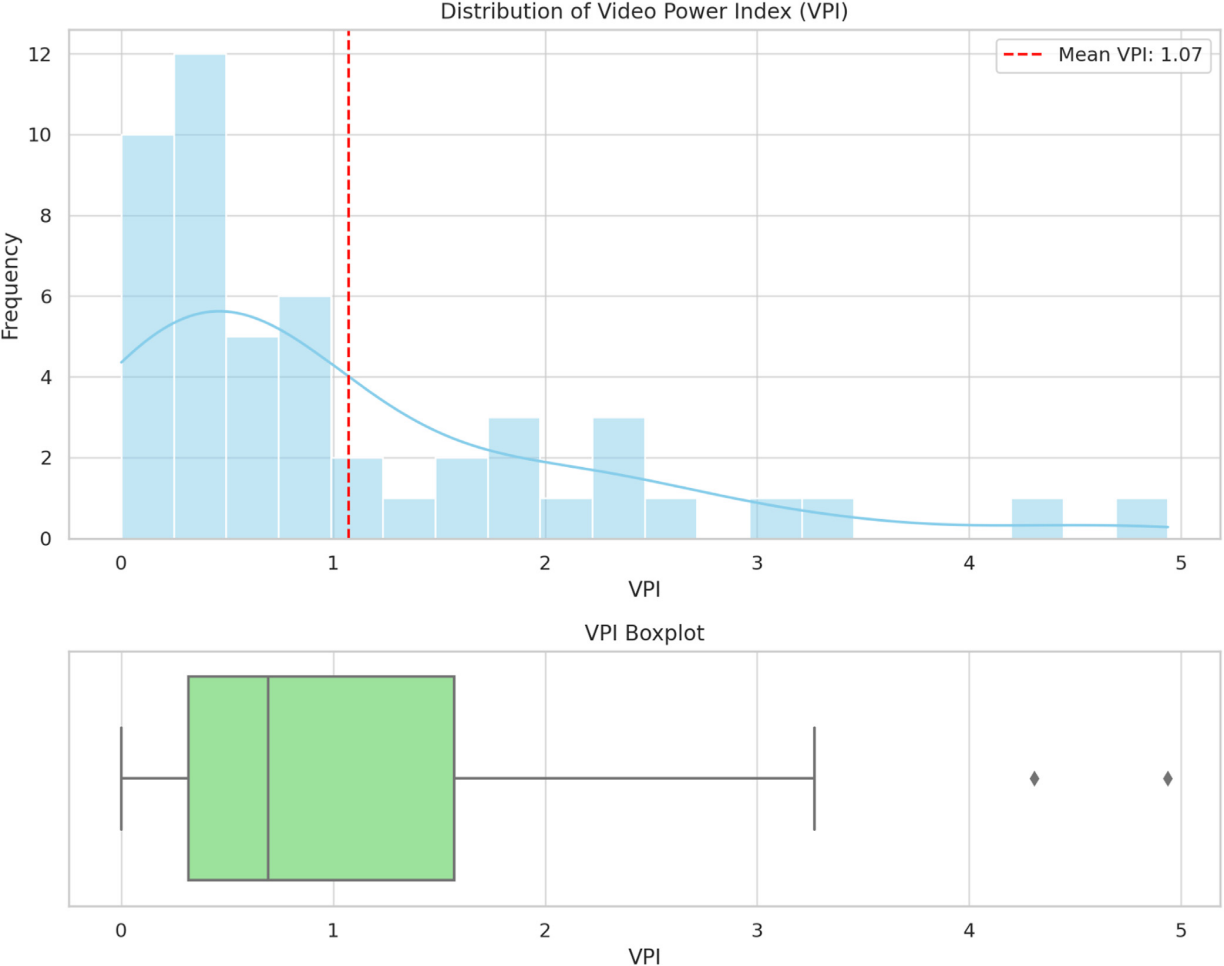
Video Power Index (VPI) distribution for YouTube cohort. A score of 0 to 0.5 indicates poor; 0.5 to 1.0, fair; 1.0 to 2.0, decent; 2.0 to 5.0, strong; and 5.0 to 10.0, excellent. The red dashed line denotes the mean VPI value of our video sample. The blue curve represents a running average for the frequency distribution.

Of the 50 videos, 24 (46.0%) were published by physicians; 20 (40.0%), by companies; and 6 (14.0%), by patients. The most prevalent type of information was an overview (44.0%); 86% of the videos were educational in nature, whereas the remaining 14% described subjective patient experiences. The mean DISCERN, JAMA, and GQS scores were 45.005 ± 1.75 (95% confidence interval [CI], 44.74-45.49), 1.825 ± 0.52 (95% CI, 1.68-1.97), and 2.97 ± 0.52 (95% CI, 2.83-3.11), respectively (Table 2). The mean Video Power Index was 1.07 ± 1.13 (Fig 2).

**Table 2:** Characteristics of Videos Included in Study (N = 50)

	Data
Video source	
Physicians	24 (46.0)
Company	20 (40.0)
Patients	6 (14.0)
Type of information	
Surgical technique	19 (38.0)
Overview	22 (44.0)
Patient experience	9 (18.0)
Video content	
Educational	43 (86.0)
Subjective	7 (14.0)
Video characteristics	
Video length, min	5.07 ± 0.21 (3.44, 6.31)
No. of views	1,624,827.44 ± 8,334.86 (1,622,540-1,627,114)
No. of likes	25,984.84 ± 1,051.76 (25,696-26,273)
No. of comments	191.62 ± 34.11 (62-321)
Months online	72.1 ± 5.29 (59.6-84.5)
DISCERN score	45.005 ± 1.75 (44.74-45.49)
JAMA score	1.825 ± 0.52 (1.68-1.97)
GQS score	2.97 ± 0.52 (2.83-3.11)

NOTE. Data are presented number (percentage) or mean ± standard deviation (95% confidence interval).

GQS, Global Quality Scale; JAMA, *Journal of American Medical Association*.

### Correlation Between Video Characteristics and Quality Scores

All the scores had no correlation with video length, months online, and number of likes. The JAMA and GQS scores were positively correlated with moderate strength with number of views; the GQS score was also positively correlated with strong strength with number of comments (Table 3).

**Table 3:** Correlations Between Scores (DISCERN, JAMA, and GQS) and Video Characteristics

	DISCERN Score		JAMA Score		GQS Score	
	ρ	*P* Value	ρ	*P* Value	ρ	*P* Value
Video length	0.24	.51	0.22	.55	0.36	.3
Months online	–0.06	.88	0.25	.49	0.16	.66
No. of views	0.3	.4	0.65	.04∗	0.68	.03∗
No. of likes	0.07	.84	0.44	.19	0.47	.18
No. of comments	0.5	.13	0.59	.08	0.81	<.01∗

GQS, Global Quality Scale; JAMA, *Journal of American Medical Association*.

∗ Statistically significant (*P* < .05).

### Comparison of Scores by Different Categories of Video Content

Videos published by physicians had greater JAMA and GQS scores than videos published by patients or companies. For all scores, overview videos had the highest quality (*P* < .05), whereas surgical technique videos had greater quality than patient experience videos for the JAMA and GQS scores (*P* < .05). Educational videos were found to have greater quality than subjective patient experience videos for all scores (*P* < .05). The details are reported in Table 4.

**Table 4:** Comparison of Quality Scores Based on Video Content and Different Categories

	Mean ± SD	Pair-wise Comparison (*P* Value)
DISCERN score		
Video source		
Physician	46.61 ± 13.4	.21 vs patient/company
Patient/company	44.12 ± 15.0	
Type of information		
Surgical technique	43.16 ± 11.3	<.01∗ vs overview .83 vs patient experience
Patient experience	38.5 ± 14.6	<.01∗ vs overview
Overview	49.37 ± 14.6	
Video content		
Educational	46.7 ± 13.9	.01∗ vs subjective
Subjective	37.1 ± 13.27	
JAMA score		
Video source		
Physician	2.23 ± 1.09	.01∗ vs patient/company
Patient/company	1.8 ± 1.04	
Type of information		
Surgical technique	1.96 ± 0.95	.03∗ vs overview <.01∗ vs patient experience
Patient experience	1.39 ± 0.96	<.01∗ vs overview
Overview	2.33 ± 1.133	
Video content		
Educational	2.12 ± 1.1	<.01∗ vs subjective
Subjective	1.34 ± 0.96	
GQS score		
Video source		
Physician	3.07 ± 1.033	.01∗ vs patient/company
Patient/company	2.7 ± 1.03	
Type of information		
Surgical technique	2.84 ± 0.73	<.01∗ vs overview .03∗ vs patient experience
Patient experience	2.1 ± 0.97	<.01∗ vs overview
Overview	3.34 ± 0.98	
Video content		
Educational	3.01 ± 1	<.01∗ vs subjective
Subjective	2.14 ± 0.9	

GQS, Global Quality Score; JAMA, *Journal of the American Medical Association*; SD, standard deviation.

∗ Statistically significant (*P* < .05).

## Discussion

The principal finding of this study was that YouTube videos on partial meniscectomy were found to be of poor to suboptimal quality. The mean DISCERN, JAMA, and GQS scores were all low, at 45.005 ± 1.75 (95% CI, 44.74-45.49; range, 15-75), 1.825 ± 0.52 (95% CI, 1.68-1.97; range, 0-4), and 2.97 ± 0.52 (95% CI, 2.83-3.11; range, 1-5) respectively. Overview videos reported some reliable information in comparison to surgical technique and patient experience videos. In addition, educational content had higher-quality information than subjective patient experience content.

Over the past decade, there has been a rapid rise in the utilization of online platforms. This has coincided with the rapid dissemination of information and high levels of interaction with online information. Among the many online tools, videos have been the primary resource for acquiring health information.^22^^,^^23^ This is corroborated by our set of analyzed videos showing decent engagement, as indicated by the Video Power Index distribution showing a mean of 1.07 ± 1.13 likes per 100 views. Moreover, there is a significant portion of orthopaedic patients who look up a diagnosis prior to going to the clinic or after an initial consultation.^24^^,^^25^ This trend can greatly affect the patient-physician relationship and influence the process of shared decision making, especially given that 38% of physicians believe pre-information bias can reduce the effectiveness of a clinic visit.^15^

A survey of current literature indicates that the quality of YouTube videos is poor in terms of acquiring medical information.^11^, ^12^, ^13^, ^14^ A previous meniscus study found a GQS score of 2.12, whereas a posterior cruciate ligament study showed a GQS score of 2.3.^16^, ^17^^,^ Our GQS score is comparable to these findings, which may be explained by a lack of an editorial process. Similarly, our JAMA score corresponds to prior reports of 2.02, 1.55, and 1.32.^16^^,^^17^^,^^26^ It is interesting to note that our DISCERN score, at 45.005, is higher than scores reported in previous studies, although it is still interpreted as not good in quality. Previous DISCERN scores of 32.2, 34.75, and 27.43 have been reported.^11^^,^^27^^,^^28^ The higher DISCERN score in our study may be attributed to the sizeable portion of videos authored by physicians (46%) and/or most videos being educational in nature (86%). In fact, Cole et al.^29^ found that physician-led YouTube videos related to anterior cruciate ligament injuries provided higher-quality educational content than other sources, which this study found as well.

A systematic review warns against the use of video characteristics such as number of views, number of likes, and recency of publication as indicators of quality and reliability.^30^ Our study corroborated this. Although there were some associations found (e.g., GQS score and number of views), there was no consistency, with a majority of tested parameters having no statistically significant associations. Thus, it appears that there still is no easy way to determine the quality of YouTube videos as a medical informational tool; the need remains for more comprehensive online resources. A possible solution could be to delineate a direct role for physicians and academic institutions in the distribution of medical YouTube videos. For example, physicians could be on advisory boards and start an initiative to create a separate catalog of approved medical information. Another possibility is to create an entirely different website, similar to VuMedi (Oakland, CA).

### Limitations

Readers should consider several limitations when interpreting the findings of this study. First, the analysis does not encompass all the content of videos pertaining to the topic of partial meniscectomy because only the first 50 videos retrieved by the keyword search were used. However, by analyzing the first 50 videos, we believe we examined the videos most commonly viewed by patients. Another limitation is that video characteristics constantly change over time, with the number of likes, number of views, and time online increasing on a daily basis. We attempted to mitigate the effect on the interpretation of our results by collecting all data within a confined period. Finally, the JAMA, GQS, and DISCERN scores are not currently validated scores but are widely used in the literature to measure the reliability, validity, and quality of online resources. Other types of analyses such as the Patient Education Materials Assessment Tool (PEMAT) could be used in future studies to better discern the role of audiovisual materials within the context of YouTube videos as an educational resource for patients.

## Conclusions

The overall quality of YouTube videos concerning partial meniscectomy remains poor to suboptimal. Currently, YouTube is not an appropriate resource for orthopaedic patients seeking information about partial meniscectomy.

## Disclosures

All authors (S.S.T., N.E.A., G.B., T.A.J., G.T.P., W.F.P.) declare that they have no known competing financial interests or personal relationships that could have appeared to influence the work reported in this paper.
